# Increased Prevalence and New Evidence of Multi-Species Chelonid Herpesvirus 5 (ChHV5) Infection in the Sea Turtles of Mabul Island, Borneo

**DOI:** 10.3390/ani13020290

**Published:** 2023-01-14

**Authors:** Dexter Miller Robben, Pushpa Palaniappan, Aswini Leela Loganathan, Vijay Kumar Subbiah

**Affiliations:** 1 Biotechnology Research Institute, Universiti Malaysia Sabah, Jalan UMS, Kota Kinabalu 88400, Malaysia; 2 Borneo Marine Research Institute, Universiti Malaysia Sabah, Jalan UMS, Kota Kinabalu 88400, Malaysia; 3 Genomics Facility, Monash University Malaysia, Bandar Sunway 47500, Malaysia

**Keywords:** fibropapillomatosis, green turtles, hawksbill, olive ridley, chelonid fibropapilloma-associated herpesvirus, ChHV5

## Abstract

**Simple Summary:**

Sea turtles worldwide are infected by a virus known as chelonid herpesvirus 5 (ChHV5), which causes fibropapillomatosis (FP). Prior to the pandemic lockdown, we conducted field sampling of 69 sea turtles in the pristine waters of Mabul Island, a diving haven located in northern Borneo. Using a molecular-based approach, we determined that the prevalence of ChHV5 in green turtles showed an increase of 42.9% compared to the previous sampling conducted in 2015–2016. Furthermore, for the first time, infection of ChHV5 in hawksbill and olive ridley turtles was also recorded in Borneo. The increased prevalence of ChHV5 should be considered as a possible threat, and efforts should be taken to mitigate the spread of the infection among sea turtles of Mabul Island and surrounding islands.

**Abstract:**

Fibropapillomatosis (FP) is a debilitating tumor disease affecting all species of sea turtles globally. The most probable etiological agent for FP is the chelonid herpesvirus 5 (ChHV5). A 2015–2016 field survey of the sea turtles at Mabul Island, Sabah, Malaysia, found three green turtles (*Chelonia mydas*) with FP tumors. However, the presence of ChHV5 was confirmed in 7.8% (9/115) green turtles and was absent (0/16) in the hawksbill (*Eretmochelys imbricata*) turtles, as determined through molecular approaches. Subsequent to this, we managed to conduct field sampling of sea turtles in November 2019, just prior to the pandemic lockdown. Here, we aim to determine the extent of ChHV5 infection, and whether the virus has spread to other species of sea turtles around Mabul Island after the first reports of ChHV5 and FP. A total of 69 tissue samples were obtained from green (63), hawksbill (5), and olive ridley (*Lepidochelys olivacea*) (1) turtles in November 2019. We observed only one green turtle that exhibited FP tumors. To determine the presence of ChHV5, viral DNA was isolated from all the tissue samples, and polymerase chain reaction (PCR) analysis targeting three highly conserved regions of the virus, i.e., the capsid protein gene, glycoprotein H gene, and glycoprotein B gene, was performed. Out of 63 green turtles, 27 were positive for the presence of the virus. The prevalence of ChHV5 in the green turtles showed an increase of 42.9% as compared to the previous sampling conducted in 2015–2016. Additionally, for the first time, three out of the five hawksbill turtles, and one olive ridley turtle, were also PCR-positive for the virus. In conclusion, this study reveals that there has been an increase in ChHV5 infection among turtles in Mabul Island over the last 3 years. ChHV5 should be considered a potential threat, and mitigation efforts should be taken to prevent the spread of infection among the endangered sea turtles of Mabul Island and surrounding islands within the Coral Triangle.

## 1. Introduction

Fibropapillomatosis (FP) is a debilitating disease of sea turtles characterized by the growth of external and internal benign tumors that infects all species of sea turtles worldwide [1,2,3]. The green sea turtle, *Chelonia mydas*, is known to be the most susceptible. The morphological changes that are caused by FP interfere with basic anatomical functions such as hydrodynamics, locomotion, and feeding, causing the affected turtles to weaken and ultimately die. FP is closely associated with chelonid herpesvirus 5 (ChHV5), also known as chelonid fibropapilloma-associated herpesvirus (CFPHV). Studies across the globe [4,5,6,7,8,9] have shown that the presence of the FP tumor is consistently linked with active infection of ChHV5. Additionally, the disease has recently become one of the major threats that contribute to the population decline in sea turtles [7,8].

There is a paucity of studies on the health status of sea turtle populations in foraging grounds. Reports of FP and ChHV5 in Asia are very limited. The first study in Taiwan, conducted in 2017, to identify ChHV5 in endangered sea turtles with FP revealed the relationship of ChHV5 in sea turtles worldwide through phylogenetic analysis [4]. ChHV5 from Taiwan’s green turtles was found to be genetically grouped with the ChHV5 from Hawaii, Puerto Rico, and Sao Tome. However, the study was limited by a sample size of only three. More experiments to expound the true relationship of the ChHV5 with the affected green turtles in Asian waters are of major interest.

The first published literature in Malaysia regarding FP dates back to 1958 [10] in the Sarawak Turtle Islands. While this was many decades ago, the data are lacking as FP was only observed and not quantified in the report. Meanwhile, the first reported occurrence of FP in Indonesia was in 1997 [11]. Here, the authors found a high prevalence of FP among green turtles at 21.5%, while no FP was found in the hawksbill turtles [11].

Evidence of the prevalence of FP and ChHV5 in green sea turtles was first documented at Mabul Island, located in northeastern Borneo [7]. The island is located at the southeastern coast of Sabah, Malaysian Borneo, and is situated strategically in the Coral Triangle region. Turtles here live and forage in the pristine waters teaming with coral reefs and seagrass, an ideal habitat for the species. The sampling period was conducted in May and November 2015 (70 sea turtles) as well as November 2016 (61 sea turtles), and it was found that 9 out of 115 green turtles were infected with the virus, and 3 of the 9 turtles had FP tumors. None of the hawksbill turtles were infected with the virus (0/16) [7]. These numbers are significantly much lower compared to what was reported earlier in Indonesia [11], but suggest that FP is spreading to turtles in non-polluted waters.

It is important that the prevalence of FP and ChHV5 are monitored over the years to obtain the current information on the problem. The present study is a follow-up to the pioneer research [7]. The results of this study provide evidence of the prevalence of ChHV5 infection among the green turtles in Mabul Island, and the first report of this infection in three species of sea turtles (green, hawksbill, and olive ridley) in a foraging population of Borneo. This study will provide a better understanding of the disease and the virus to develop a reliable approach to control and minimize the risk of the disease. The findings will also provide information for conservationists for monitoring purposes and efforts in maintaining a healthy population of sea turtles.

## 2. Materials and Methods

### 2.1. Sample Collection

The study site was situated in Mabul Island, Semporna, (4.25° N, 118.63° E), located in the southeastern coast in Sabah, Malaysia. The island is small and oval, bordered by sandy beaches on the northwest corner of a larger 200-hectare reef [12]. Sample collection was carried out for four days in November 2019. The sea turtles were hand-caught underwater at established dive sites of the island, then photographed, measured, and tagged on both anterior flippers with Inconel flipper tags. The straight carapace length (SCL) of each sea turtle was measured with 1.0 m Mitutoyo stainless vernier calipers (accurate to 0.05 ± 0.15 mm), measuring from the anterior point at the midline (nuchal scute) to the posterior end of the supracaudal region [13].

We collected normal (non-tumor) skin and tumor nodules of the sea turtles. The normal skin tissue samples were taken with a biopsy punch from all the turtles. Proper aseptic technique was observed. The first step in the sampling procedure was to disinfect the sampling tissue with 70% alcohol. After disinfection, normal skin was removed from the flippers of the sea turtles using a 5 mm or 8 mm diameter biopsy punch. If any FP tumors were visible on any parts of the sea turtle, the sample was surgically removed with a scalpel and blade. Disinfection using Betadine was applied after removal of the biopsy punch. The turtles were then released back into the sea after the sampling procedure was completed safely [14].

### 2.2. DNA Isolation and Polymerase Chain Reaction (PCR) of ChHV5 Regions

Genomic DNA of the samples was extracted using the Wizard^®^ Genomic DNA Purification Kit (Promega, Madison, WI, USA) following the manufacturer’s instructions. The extracted DNA was then used for ChHV5 detection by PCR. PCR assays were performed for the detection and screening of the ChHV5 from the samples using three independent primer sets amplifying the highly conserved regions of the ChHV5, namely, the capsid protein gene UL18, glycoprotein H gene UL22, and glycoprotein B gene UL27 (Table 1).

These three sets of primers were developed and found to be more sensitive than the other available oligonucleotides primers shown in a previous study [15]. Amplification of ChHV5 gene regions was carried out using the GoTaq Flexi Kit (Promega, USA) with 20 µL volumes containing 50 ng of genomic DNA, 0.2 U of *Taq* DNA polymerase, 1× buffer, 2.0 mM of MgCl_2_, 15 pmol of each primer, and 0.4 mM of dNTPs in a PT1000 thermocycler (Bio-Rad, Foster City, CA, USA) with the following PCR conditions: initial denaturation at 94 °C for 1 min, followed by 35 cycles of denaturation at 94 °C for 30 s, optimal annealing temperature for 30 s, extension at 72 °C for 30 s, followed by a final elongation step at 72 °C for 7 min [15]. After PCR amplification, 1.5% agarose gel electrophoresis was performed to verify whether the amplicons were at the expected sizes.

### 2.3. Sanger Sequencing and Phylogeography of the ChHV5 Region

PCR products were cloned to produce TOPO TA cloning vectors (Invitrogen, Waltham, MA, USA) according to the manufacturer’s protocol. The plasmids were then isolated using the GeneJet Plasmid Miniprep Kit (Thermo Scientific, Waltham, MA, USA). Next, Sanger sequencing of the plasmid DNA was performed to confirm the identification of ChHV5 in the samples. The reaction was set up using the BigDye Terminator Kit v3.1 using both the forward and reverse primers, and analyzed on an ABI 3130 DNA sequencer (Applied Biosystems, Waltham, MA, USA.). The DNA sequences of the ChHV5 region were compared with available sequences in GenBank (https://www.ncbi.nlm.nih.gov/genbank, accessed on 12 February 2021) for verification of the DNA identity using BLAST (basic local alignment search tool). Following multiple sequence alignment using ClustalW, phylogenetic trees were constructed using the maximum likelihood (ML) method based on Tamura’s three-parameter model with MEGAX [16]. Bootstrapping with 1000 reiterations was performed to test the reliability of clustering patterns.

### 2.4. Ethics Statement

Approval from the Universiti Malaysia Sabah Animal Ethics Committee (JEHUMS) was obtained, which allowed the study to be conducted on the sea turtles of Mabul Island. The animal ethical clearance number is UMS/PPPI1.3.2/800-2/1/17 Jilid 4 (06).

## 3. Results and Discussion

A total of 69 sea turtles comprising 63 green turtles (SCL ranging from 35.6 to 97.1 cm), 5 hawksbill turtles (SCL ranging from 42.7 to 63.9 cm), and 1 olive ridley turtle (SCL 54.1 cm) were caught and sampled at Mabul Island. Thus, there was a higher number of green turtles that were captured as compared to the other two species. The results show that the dominant species of sea turtle in Mabul Island is the green turtle, followed by the hawksbill turtle [12].

**Figure 1:**
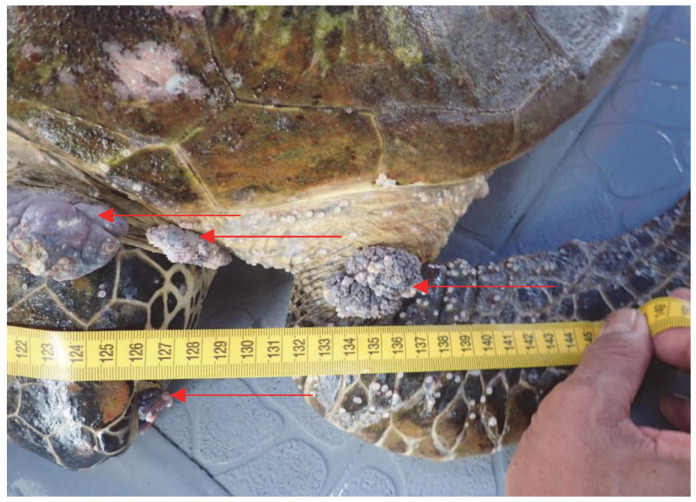
A juvenile green sea turtle with multiple FP tumors (arrows) on its flippers, neck, and eyes, that was caught on Mabul Island during the sampling period in November 2019.

**Figure 2:**
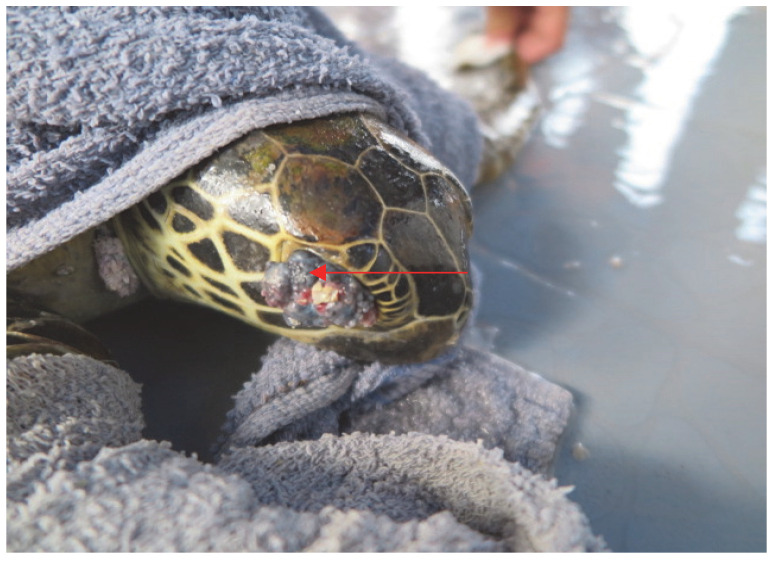
A juvenile green sea turtle with FP tumors (arrow) on its eyes, a condition known as corneal fibropapilloma, causing the affected sea turtle to be visually impaired.

The capture of only one olive ridley turtle suggests that Mabul Island is a foraging ground for the species, although sightings of this turtle in Mabul Island are rare. Most of the sea turtles captured during the sampling period appeared healthy except for one green turtle (Figure 1 and Figure 2). This green turtle was found to exhibit multiple nodules of FP on the flippers, neck, and the eyes (condition known as corneal FP). The tumors can adversely affect sea turtles and disrupt their locomotion, feeding, vision, and organ function. The disruptions will weaken the turtles, and eventually lead to immunosuppression or death [17,18].

Following DNA extraction, all 69 sea turtles were screened for ChHV5 using PCR by amplifying three target regions of their respective viral genes. The viral genes, namely, the capsid protein gene (UL18), glycoprotein H gene (UL22), and glycoprotein B gene (UL27), were chosen as they are highly conserved genes of the herpesvirus. Out of 69 sea turtles (68 clinically healthy sea turtles (no tumors observed) and 1 FP-exhibiting sea turtle), 31 individuals were PCR-positive for ChHV5 (Table 2). Out of the 31 sea turtles, 26 individuals were positive for the capsid protein gene UL18. For the glycoprotein B gene UL27, 11 out of 31 individuals were positive. There was no detection of the glycoprotein H gene UL22 in any of the individuals (see Supplementary Table S1).

The prevalence of ChHV5 in the green turtles increased to 42.9% as compared to the previous sampling conducted in 2015–2016, at which point it was only 7.8%; however, the sample sizes are different (115 green turtles in the previous study). Similar results were reported in Anguilla [19], where the number of green turtles exhibiting tumors increased from one individual in 2010 to 31% of the turtles caught in 2014. It was reported that 17% of the green turtles caught in Anguilla had tumors in 2015. However, precise details of the study were not reported. In addition, the virus was not detected in the 16 hawksbill turtles caught in that study [20].

Additionally, three out of the five hawksbill turtles and one olive ridley turtle were also PCR-positive for ChHV5 in the present study. The study conducted in 2015–2016 [7] had no viral detection in hawksbill turtles. Therefore, this is the first evidence of ChHV5 infection in more than one species of sea turtle (i.e., hawksbill and olive ridley) in Mabul Island. Although ChHV5 infections are mostly reported in green turtles globally due to its panzootic status [19], it is also noted that all species of sea turtles have been recorded with ChHV5 [1]. The first reported case of FP in the hawksbill turtle was from Brazil. The FP tumors were collected, and histopathology analysis showed features that met the criteria of typical FP. However, no molecular analysis detecting the ChHV5 viral DNA was performed [21].

Another related study conducted on hawksbill turtles from Kuwait reported the detection of ChHV5 through molecular techniques [22]. However, the six hawksbill turtles that were PCR-positive for the virus were clinically healthy turtles, similar to those reported in the present study. These findings suggest persistent latent infections of ChHV5 in seemingly healthy turtles without detectable tumors. On the other hand, the detection of ChHV5 in an olive ridley turtle was reported in Texas, USA, where it was found dead with a large FP tumor observed on its right front flipper. A PCR assay was performed, and the tumor was found positive for ChHV5. This was further verified through sequencing analysis [23].

In this study, all of the FP-exhibiting sea turtles were found to be PCR-positive for ChHV5. Koch’s postulate states that for establishing causation, the virus must be isolated from a diseased organism, which is then grown in pure culture, and the development of disease occurs when the virus is re-introduced into a healthy organism [22]. ChHV5 will grow in vitro if the complex, three-dimensional structure of the turtle skin is replicated [24]. This finding shows a realistic picture of ChHV5 replication, which is crucial to understand the virus and eventually fulfil Koch’s postulate [24]. Although Koch’s postulate is yet to be fulfilled, the ChHV5 is identified as the most probable etiologic agent of fibropapillomatosis (FP), as numerous studies have linked ChHV5 with FP, whereby the virus has been consistently detected via PCR [4,6,7].

In addition, 30 individuals of clinically healthy sea turtles in the current study were also PCR-positive for ChHV5. This shows that although the sea turtles are not manifesting the FP tumor, ChHV5 may still be present. There are many theories that have been suggested for this. The most accepted theory is the ability of ChHV5 to cause latent infections, a common characteristic of the herpesvirus family [22,25]. The virus is considered to be present in sea turtles if any of the regions are successfully amplified through PCR. This is due to the efficiency of the detection in a latently infected sea turtle that requires a more sensitive PCR assay. Hence a combination of more than one gene is used for the screening of the virus, especially in a clinically healthy sea turtle [15].

As there were no positive amplifications of the UL22 region, only UL18 and UL27 regions were subjected to phylogenetic analysis. Five positive amplicons for the UL18 region and one positive amplicon for the UL27 region were selected randomly and subjected to cloning. These positive amplicons originated from the green and hawksbill turtles (i.e., one amplicon from hawksbill turtle sample and five amplicons from green turtle samples). The positive white colonies, which contain the target region, were then selected for plasmid extraction. The extracted plasmid DNA was then used as a template for Sanger sequencing. The sequences obtained were compared with other sequences available in the Genbank database, using BLAST search to verify the sequence identity of the amplicons. Since the sequences for UL18 from the five samples were identical, no other samples were sequenced. All the ChHV5 DNA sequences obtained in the present study were deposited in Genbank with the accession numbers OQ189658 to OQ189663.

**Figure 3:**
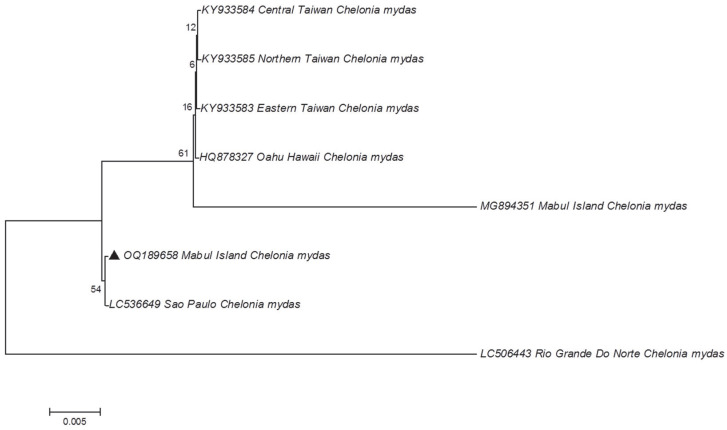
Phylogenetic tree of ChHV5 partial major capsid protein variants inferred using the maximum likelihood method (Tamura’s three-parameter model). The sample from this study is indicated with a black triangle.

**Figure 4:**
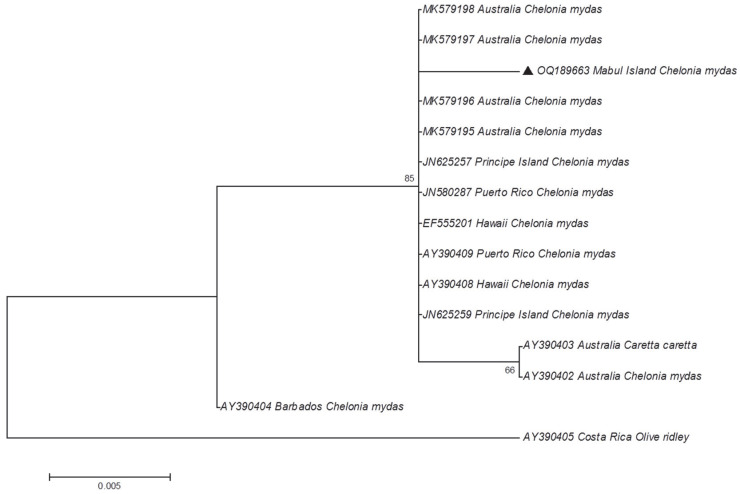
Phylogenetic tree of ChHV5 partial glycoprotein b variants inferred using the maximum likelihood method (Tamura’s three-parameter). The sample from this study is indicated with a black triangle.

The homology search results showed that the sequences obtained were the ChHV5 regions, as expected. Additionally, phylogenetic analysis that was constructed using the maximum likelihood (ML) method for the glycoprotein B gene (UL27) and the capsid protein gene (UL18), individually (Figure 3 and Figure 4). For UL18, only one representative sequence (OQ189658) was used since all the others were identical.

The phylogenetic tree reveals that the ChHV5 Mabul Island isolates are clearly grouped with other ChHV5 isolates from other regions, verifying the identity of the Mabul Island isolates. Furthermore, there are no clear geographical clusters of the ChHV5 isolates, indicating that the ChHV5 Mabul Island isolates are similar to different isolates from different regions such as Brazil, Florida, Hawaii, and Puerto Rico. We also noticed that for the UL18 sequence, the newly obtained sample from Mabul (OQ189658) was not grouped with the ChHV5 strain (MG894351) that was obtained in 2015. This information suggests that there is no clear demarcation of the ChHV5 lineage based on the geographical regions, as was also observed in Li et al. [4]. However, this must be further verified since the sequence obtained for UL18 is relatively short. A longer region ought to be analyzed (or whole viral genomes) to determine the strain or lineage of the ChHV5 detected in the samples collected from Mabul Island. Nevertheless, if the ChHV5 is in fact from a common strain, this could be due to the wide range of movements (i.e., migration) of the sea turtles, as reported previously elsewhere [7,26,27]. Several studies have examined the possible route of transmission of ChHV5 [27,28,29]. Three types of samples (skin tissue, tumor tissue, and swab samples (ocular, nasal, and cloacal)) were collected to study the transmission of the virus [27]. The results revealed that the ChHV5 DNA was detected in the swab samples, which supports the possibility of a horizontal transmission route of the virus through the excretion and secretion of bodily fluid [27]. The migratory nature of sea turtles begins when the turtle hatchlings undertake long-distance migrations, following oceanic currents and eventually settling down in coastal waters as juveniles [30]. The migration of adult turtles can span thousands of kilometers between breeding/nesting grounds and foraging grounds. Hence, healthy sea turtles may come into contact with infected sea turtles during this long-distance migration period, or when they congregate at foraging grounds where they spend long periods of time [31]. A study conducted in Queensland, Australia, showed that the six distinct variants of ChHV5 were associated with host foraging grounds, and no association was found between host genetic origins. Thus, these findings further support the horizontal transmission of ChHV5 among sea turtles at foraging grounds, and less likely at rookeries [29]. Moreover, it is also believed that marine leeches (*Ozobranchus* spp.) play a role in the distribution of the ChHV5 worldwide [32,33,34,35]. A study on parasites affecting sea turtles in Hawaii [32] found that the *Ozobranchus margoi* carries high viral DNA loads. This finding seems to suggest the role of marine leeches as a mechanical vector for ChHV5 [32]. Therefore, the global distribution of the ChHV5 can be explained by the long-distance migration period of the sea turtles and the role of marine leeches as the mechanical vector for ChHV5. However, no marine leeches were observed on the captured sea turtles in the present study.

## 4. Conclusions

This study confirms the increased prevalence of ChHV5 in green turtles at Mabul Island after 3 years, and new evidence of ChHV5 infection in hawksbill and olive ridley sea turtles in this region. The study highlights the detection of ChHV5 in sea turtles of Mabul Island through molecular techniques, and phylogenetic analysis was performed to facilitate the understanding of the distribution of the virus isolate in the study area with other isolates from different geographical regions.

FP should be considered as one of the major threats to the population of sea turtles in Mabul Island, as the turtles with FP often do not survive. In addition, ChHV5 needs to be considered as an emerging virus that threatens the sea turtle populations in Mabul Island. It is imperative that proper actions and efforts are taken for the conservation of sea turtle populations. A follow-up study should be conducted to re-evaluate the status of the prevalence of ChHV5 and FP in the sea turtle populations included in this study. Additionally, more studies are needed in neighboring regions to gather more information on the spread of FP and ChHV5.

## Figures and Tables

**Table 1:** Sequences of the oligonucleotide primers for amplifying the ChHV5 regions and expected amplification size (bp).

Region	Primer Name	Primer Sequences 5′–3′	Size (bp)
Capsid protein gene	UL18	F: GTGGAACCCCGCCGGGTAAT R: TGATCCGGGCCGAGTAGCGC	140
Glycoprotein H gene	UL22	F: AACGCCCTTTCCTCCGACCCATATT R: GCTGGGGGAGCATCGTGCAAA	179
Glycoprotein B gene	UL27	F: CTAGATACATACTGGCCRTGCTCGTC R: GCCAGCGACCATCCGGAG	143

**Table 2:** Detection of ChHV5 in tissues with and without FP tumors in the green, hawksbill, and olive ridley turtles.

Turtle Species	No. of Samples	Turtles with FP Tumor (PCR-Positive)	Turtles without FP Tumor (PCR-Positive)
Green	63	1	26
Hawksbill	5	0	3
Olive Ridley	1	0	1
TOTAL	31		

## Data Availability

The data presented in this study are available on request from the corresponding author. The newly obtained DNA sequences are available at GenBank with the accession numbers OQ189658 to OQ189663.

## Supplementary Materials

The following supporting information can be downloaded at https://www.mdpi.com/article/10.3390/ani13020290/s1. Table S1: Positive-PCR sea turtle samples according to the three regions of ChHV5 used in this study (i.e., UL18, UL22, and UL27).
